# Neuroarthropathy of the hip following spinal cord injury

**DOI:** 10.4103/0019-5413.73665

**Published:** 2011

**Authors:** Bibek Banskota, Binod Bijukachhe, ShresthaBabu Kazi, Ashok K Banskota

**Affiliations:** Department of Orthopaedics and Trauma, B and B Hospital, Kathmandu University, P.O. Box-2481, Nepal

**Keywords:** Charcot’s hip, neuroarthropathy, spinal cord injury

## Abstract

We present the case of a 33-year-old male who sustained a burst fracture D12 vertebrae with spinal cord injury (ASIA impairment scale A) and a right mid-diaphysial femoral shaft fracture around 1.5 years back. The patient reported 1.5 years later with a swelling over the right buttock. Arthrotomy revealed serous fluid and fragmented bone debris. The biopsy showed a normal bony architecture with no evidence of infection and malignant cells. Hence, a diagnosis of Charcot’s hip was made. Charcot’s neuroarthropathy of the feet is a well-recognized entity in the setting of insensate feet resulting from causes such as diabetes or spina bifida. Although Charcot’s disease of the hips has been described, it is uncommon in association with spinal cord injury, syphilis and even with the use of epidural injection. The present case highlights the fact that neuroarthropathy of the hip can occur in isolation in the setting of a spinal cord injury, and this can lead to considerable morbidity.

## INTRODUCTION

Charcot’s neuroarthopathy is a well-recognized entity in the setting of absent sensation in the foot, spine, shoulder, wrist, knee and other joints.^1^–^6^ It is a challenging problem to treat because of its chronicity and its indolent nature. Even though a well-recognized possibility, to the best of our knowledge, there has been no report in the English literature on the isolated involvement of a hip joint by Charcot’s neuroarthropathy in a patient with spinal cord injury.We present the case of a young man with spinal cord injury who developed a neuroarthropathic right hip that lead to a massive swelling, impending decubitus ulcer and difficulty in transferring and ambulation in a wheelchair.

## CASE REPORT

**Figure 1:**
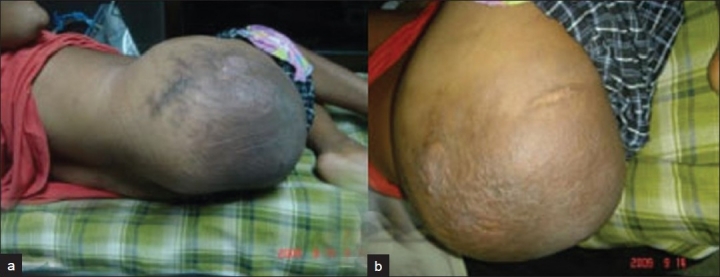
Clinical photographs (a,b) showing massive boggy swelling of the right hip and buttock with extensive bruising and impending pressure sore

**Figure 2:**
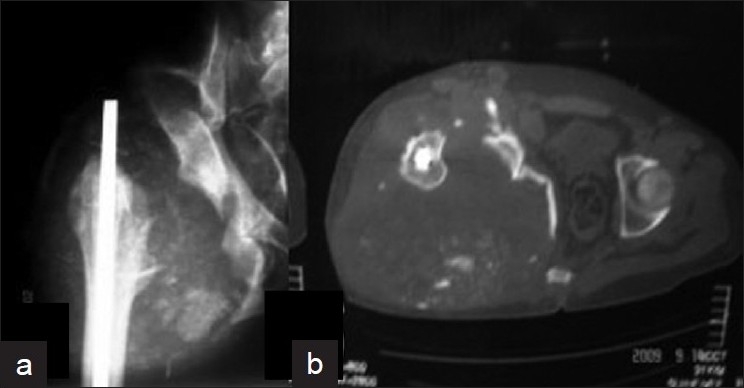
Anteroposterior radiographs (a) and computed tomography scan of hip (b) at time of presentation showing dramatic destruction of the right hip joint with fragmented bone debris in place

A 33-year-old male presented with a huge painless swelling of the right hip and buttock region. He sustained road traffic accident (RTA) 1.5 years ago leading to fracture of the 12^th^ thoracic vertebra with complete paraplegia (ASIA impairment scale A) and an open right mid-diaphyseal femur fracture. He was treated conservatively for fracture spine and with a Kuntscher nail for fracture femur. On clinical examination there was a swelling measuring 30 cm × 30 cm on his right hip and buttock. The swelling was cystic, boggy and variegated in consistency. The overlying skin was extensively bruised and a pressure sore was imminent [Figure 1]. Although painless, he however felt discomfort while sitting. On neurological examination the patient was still ASIA A. X-rays and CT scan showed complete destruction of the femoral head and neck with a large effusion and fragmented bone debris in place of the native hip joint [Figure 2].

**Figure 3:**
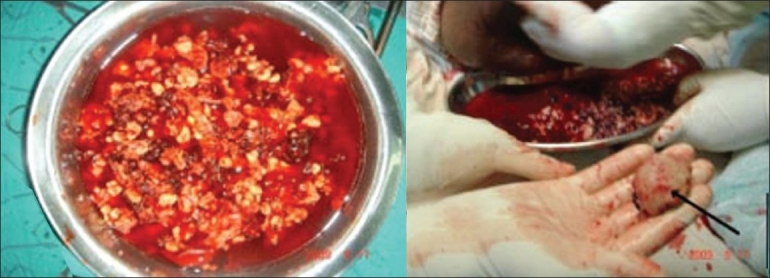
Intra-operative photograph of decompression showing serohemorrhagic fluid, fragmented bone debris and remnant of femoral head (arrow)

Aspiration from swelling revealed straw-colored serous fluid. A biopsy was planned to rule out the possibilities of infection or malignancy and to establish the diagnosis. Fragmented bone pieces and serous fluid were found intra-operatively [Figure 3]. There was no pus. The fragmented bone debris was removed and a Girdlestone arthroplasty was performed. As the femoral shaft fracture had already united, the Kuntcher nail was also removed. The wound was closed over a corrugated drain to facilitate drainage of the residual fluid and debris. The drain was removed after 7 days as the discharge was minimal by then.

**Figure 4:**
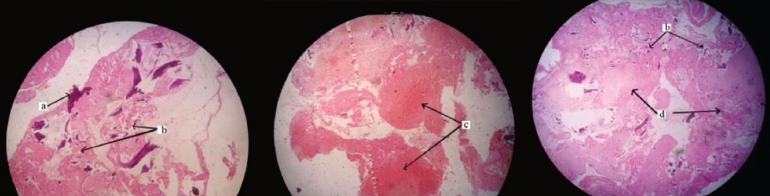
Histopathological sections showing multiple fragments of (a) necrotic bone, (b) calcification and (c) hemorrhage with (d) aggregates of fibrous debris

Histopathological findings revealed multiple fragments of necrotic bone, calcification and hemorrhage with aggregates of fibrous debris [Figure 4]. Culture of the specimen was negative for any microorganisms. Based on the history, clinical, radiological and histopathological findings, a diagnosis of Charcot’s neuroarthropathy of the right hip joint secondary to spinal cord injury was reached.

At the last follow-up, 6 months following decompression, there was still some serous discharge from the proximal 0.5 cm of the surgical wound that had failed to heal. The culture report of this discharge was negative for any microorganisms. This wound and the discharge eventually settled with regular local dressing and the patient was able to sit and transfer without any discomfort.

## DISCUSSION

Joint neuroarthropathy was first described by Jean Marie Charcot, a French neurologist, in patients with tertiary syphilis.^1^ Etiology includes diabetes mellitus, spina bifida, spinal cord injury and less common causes like tumors, congenital insensitivity to pain and epidural injection use affecting joints in both the axial and the appendicular skeleton.^2^–^5^,^7^–^12^ The neurotraumatic theory^13^ suggests that repetitive microtrauma in an insensate area triggers arhtropathy whereas the neurovascular theory^14^ implicates loss of autonomic sympathetic tone leading to persistent hyperaemia, osteoclast activation and bone resorption as the trigger. It is likely that both these phenomenon contribute to the eventual clinical picture that we know as Charcot’s neuroarthropathy.

Most reports on neuroarthropathy focus on the foot and ankle,^15^–^17^ mainly in patients suffering from diabetes mellitus. Sobel *et al*. reported on five cases of Charcot’s neuroarthopathy in patients with Spinal Cord Injury (SCI), all of them affecting the spine; three of these patients had complete paraplegia and two had complete tetraplegia.^12^ They implicated surgical laminectomy and the ensuing instability as a possible cause of neuropathic psuedarthrosis in four of the five cases and advocated repeat surgical stabilization and fusion for improved outcomes. Shem reported on a 46-year-old man with a 30-year history of T4 paraplegia secondary to an ependymoma who gradually developed painless swelling and arthropathy of his left wrist that was managed conservatively with bracing.^4^ The author indicated that such an occurrence in the setting of SCI can compromise wheelchair use and performance of activities of daily living independently.

The major sensory nerve supply to the hip comes from the obturator nerve, with important contributions from the femoral nerve, superior gluteal nerve, sciatic nerve and the nerve to quadratus femoris^18^ and therefore any lesion higher than the origin of these nerves would render the hips insensate. Affliction of the hips by Charcot’s neuroarthopathy has been reported in isolated case reports or small series in etiologies ranging from diabetes mellitus,^19^ tabesdorsalis,^20^ chondrosarcoma of the cervical spine,^10^ congenital insensitivity to pain,^11^ intra-articular steroid use^21^ and even the use of epidural anesthesia in total joint arthroplasty.^22^

We believe that this differentiation in etiology of a neuropathic hip is important because the patients with motor power may do well with total hip arthroplasty, although this is known to be fraught with its own complications,^23^ whereas a Girdlestone procedure or just symptomatic treatment may be the only option in patients with complete SCI, as in our case. Knee effusions and, less commonly, hip joint effusions and heterotopic ossification have been noted by different authors in the setting of SCI.^6^ Repetitive microtrauma has been implicated in the etiology but the significance of such a finding and whether it precedes heterotopic ossification and possibly arthropathy is still unclear.

How and when to screen for development of neuroarthropathy of the hip in a patient with SCI is a question open to debate. Patients with SCI are already much compromised in their ability to ambulate. Occurrence of hip neuroarthropathy in them can severely compromise their ability for wheelchair ambulation and transfer because the resultant effusion and swelling can be massive and cause skin breakdown. We feel that early recognition and a Girdlestone arthroplasty of the affected hip can mitigate this problem. In any case, awareness of this problem in the setting of SCI can prevent delays in management and the development of pressure sores and ulcers, which are a menace to manage and incur substantial resources, both professional and financial, which are both scarce in our setting of a rural majority and a socioeconomically constrained population.
